# Malignancy risk score in thyroid nodules with atypia of undetermined significance cytology: an approach based on cytological subgrouping and ultrasonographic data

**DOI:** 10.20945/2359-4292-2026-0092

**Published:** 2026-08-12

**Authors:** Yusuf Öztürk, Naile Kökbudak, Muhammet Kocabaş, Melia Karaköse, Mustafa Kulaksizoğlu, Feridun Karakurt

**Affiliations:** 1 Sakarya University Training and Research Hospital, Department of Endocrinology and Metabolism, Sakarya, Turkey; 2 Department of Pathology, Necmettin Erbakan University Meram Faculty of Medicine, Konya, Turkey; 3 Department of Endocrinology and Metabolism, Necmettin Erbakan University Faculty of Medicine, Konya, Türkiye

**Keywords:** Atypia of undetermined significance, thyroid nodule, ultrasonography, nuclear atypia, AUS-malignancy risk score

## Abstract

**Objective:**

Thyroid nodules diagnosed as atypia of undetermined significance (AUS) are one of the most challenging categories in thyroid cytopathology due to histopathological ambiguity and complexities in clinical management. Therefore, this study aimed to develop and evaluate the diagnostic accuracy of a novel malignancy risk scoring system integrating cytological subclassification and key ultrasonographic features.

**Subjects and methods:**

A retrospective analysis was conducted on 357 patients who underwent thyroidectomy following an AUS cytology diagnosis. Cytological samples were subclassified as AUS-N or AUS-O, based on the presence of nuclear atypia, and fundamental sonographic parameters were recorded. Independent predictors of malignancy were identified using multivariable logistic regression, and the AUS-malignancy risk score (AUS-MRS) was developed accordingly. Diagnostic performance was compared with existing classification systems: ACR-TIRADS, EU-TIRADS, and K-TIRADS.

**Results:**

The AUS-MRS demonstrated the highest diagnostic performance, with a sensitivity of 84.6%, specificity of 79.6%, and a diagnostic odds ratio (DOR) of 21.8. Among the conventional systems, ACR-TIRADS showed the highest sensitivity (89.1%) but had lower specificity (69.7%) and a DOR of 18.4. EU-TIRADS and K-TIRADS showed more balanced metrics, with sensitivities of 82.7% and 81.4%, specificities of 73.6% and 74.1%, and DORs of 18.2 and 16.7, respectively. The inclusion of nuclear atypia increased specificity across TIRADS models (>85%); however, this was accompanied by reduced sensitivity and did not yield substantial gains in diagnostic performance.

**Conclusion:**

The AUS-MRS may offer improved diagnostic utility compared to existing ultrasound-based classification systems for assessing malignancy risk in AUS nodules.

## INTRODUCTION

Fine-needle aspiration biopsy (FNAB) remains the primary diagnostic tool for assessing malignancy risk in thyroid nodules (^1^). The Bethesda System for Reporting Thyroid Cytopathology (TBSRTC), most recently revised in 2023, standardizes thyroid cytology reporting into six diagnostic categories to guide clinical management (^2^). Among these, atypia of undetermined significance (AUS) poses particular diagnostic challenges due to histopathological heterogeneity and the complexity of subsequent clinical decision-making. Recommended management options for AUS include repeat FNAB, molecular testing, or diagnostic thyroidectomy; however, the high costs of molecular tests and the invasive nature of surgery often limit their routine application (^1^,^2^). In this context, ultrasonography (US) plays a central role in both the initial evaluation and ongoing management of thyroid nodules.

Previous studies have demonstrated that various US features may assist in malignancy risk stratification for nodules diagnosed as AUS, although no single sonographic feature is sufficiently reliable in isolation (^3^,^4^). This limitation led to the development of ultrasound-based risk stratification systems, most notably, the Thyroid Imaging Reporting and Data System (TIRADS), which integrate multiple sonographic features into composite risk scores and are implemented in widely used frameworks, including the American College of Radiology (ACR-TIRADS), the European Thyroid Association system (EU-TIRADS), and the Korean TIRADS (K-TIRADS) (^5^–^8^). Despite widespread clinical adoption, the diagnostic performance of these systems in AUS nodules remains moderate, as highlighted in prior studies and meta-analyses (^9^–^12^).

The heterogeneity of the AUS category, coupled with widely variable reported malignancy rates ranging from 17 to 83%, has prompted efforts to improve risk stratification through cytological subclassification (^13^,^14^). Various studies have shown that nuclear atypia is associated with a higher malignancy risk compared with architectural atypia (^15^–^18^). Accordingly, the 2023 third edition of TBSRTC introduced the subclassification of AUS with nuclear atypia (AUS-N) and AUS with other atypia (AUS-O) to refine risk assessment (^19^). Nevertheless, cytological subclassification or sonographic assessment alone may be insufficient for accurate malignancy prediction.

Therefore, integrating cytological subgroups with key ultrasonographic features may offer a more comprehensive approach to malignancy risk stratification for AUS-diagnosed nodules. Accordingly, we aimed to develop and validate the AUS-Malignancy Risk Score (AUS-MRS), a novel malignancy risk scoring system that combines cytological subclassification with sonographic parameters. In addition, the diagnostic performance of AUS-MRS was compared with three widely used TIRADS-based systems: ACR-TIRADS, EU-TIRADS, and K-TIRADS.

## SUBJECTS AND METHODS

### Study design and patient selection

This retrospective observational study was conducted through collaboration between the Division of Endocrinology and Metabolism and the Department of Pathology at Necmettin Erbakan University Faculty of Medicine (Turkey) from 2016 to 2025. The study protocol was approved by the institution’s ethics committee (decision no. 2024/5399; date: 20/12/2024). Patients included in the analysis had an initial FNAB diagnosis of AUS based on the TBSRTC and subsequently underwent thyroidectomy. Demographic data (e.g., age and sex), US characteristics, cytologic results, and final histopathological findings were retrospectively retrieved via the institutional hospital information system.

All AUS cytology slides were re-evaluated according to the updated 2023 TBSRTC criteria by an experienced pathologist blinded to the final histopathological diagnosis. Cases not confirmed as AUS upon re-evaluation were excluded. Confirmed AUS cases were subclassified into two groups: AUS-N and AUS-O. Patients were also excluded if their US reports lacked sufficient detail about nodule features, or if there was a mismatch in nodule localization between US and surgical pathology reports. Ultimately, 357 patients who met all eligibility criteria were included.

### Ultrasound evaluation and classification

Prior to FNAB, ultrasound images were obtained by endocrinologists with at least 10 years of experience. A 12L3 transducer (3.6–12.9 MHz) was used with a Siemens Healthineers Acuson Juniper ultrasound system (Berlin, Germany). Each nodule was evaluated for size, composition, echogenicity, shape, margin characteristics, and echogenic foci. Nodule composition was classified as cystic, spongiform, mixed cystic-solid, or solid. Echogenicity was defined as anechoic, isoechoic, hyperechoic, mildly hypoechoic, or markedly hypoechoic, relative to the surrounding thyroid parenchyma. Shape was classified as wider-than-tall or taller-than-wide. Margins were categorized as smooth, lobulated, irregular, or showing extrathyroidal extension. Echogenic foci were characterized as comet-tail artifacts, macrocalcifications, peripheral calcifications, or microcalcifications. Based on previous research and the study design, solid composition, mild or marked hypoechogenicity, a taller-than-wide shape, irregular margins or extrathyroidal extension, and the presence of peripheral or microcalcifications were considered suspicious for malignancy. Each nodule was scored using the criteria of the ACR-TIRADS (^1^–^5^), EU-TIRADS (^2^–^5^), and K-TIRADS (^2^–^5^) classification systems, and corresponding risk categories were documented.

### Fine-needle aspiration and histopathological assessment

FNAB specimens were prepared using the conventional smear technique. After aspiration, approximately 1–2 drops of material were expelled onto a clean glass slide. A second slide was then placed over the first at an angle of approximately 45–90° and gently drawn across to produce a thin, even smear, similar to the peripheral blood smear technique. According to the staining protocol, smears were air-dried or immediately fixed. To ensure adequate sampling, at least two aspirations were performed for each lesion. Histopathological diagnoses were made in accordance with the 2022 World Health Organization Classification of Thyroid Neoplasms (^20^). Nodules were categorized as benign or malignant. Noninvasive follicular thyroid neoplasm with papillary-like nuclear features, due to its indolent biological behavior, was classified as benign.

### Development of the AUS-MRS

Based on the results of the multivariate logistic regression analyses, the novel malignancy risk scoring system (i.e., AUS-MRS) was developed. This system integrated cytological subclassification and key ultrasonographic parameters identified as independent predictors of malignancy.

### Statistical analyses

All statistical analyses were performed using jamovi (v. 2.6.4) and R (v. 4.5.0). Continuous variables were presented as mean ± standard deviation or median and interquartile range; categorical variables were summarized as frequencies and percentages. Comparisons between benign and malignant cases were performed using the chi-square or Fisher’s exact test for categorical variables, and the independent samples t-test or Mann-Whitney U test for continuous variables, as appropriate for data distribution. Initially, univariate analyses identified candidate predictors of malignancy. Variables with *p*-values < 0.10 were included into a multivariable binomial logistic regression model with backward elimination to identify independent predictors. Collinearity was assessed via variance inflation factors, and model performance was evaluated using Nagelkerke’s R^2^. The AUS-MRS was developed from the final regression model using normalized β coefficients. Diagnostic performance of AUS-MRS and other classification systems (i.e., ACR-TIRADS, EU-TIRADS, and K-TIRADS) was assessed with receiver operating characteristic (ROC) curve analysis. The area under the curve (AUC), 95% confidence intervals, and optimal cut-off points (determined by the Youden index) were reported. Pairwise comparison of AUCs utilized the DeLong test for correlated ROC curves. Internal validation was conducted using a split-sample approach, allocating 70% of the dataset for model development and 30% for validation. Sensitivity, specificity, positive and negative likelihood ratios (LR+ and LR−), and diagnostic odds ratios (DOR) were calculated for each scoring system. Pairwise agreement among classification systems was assessed using Cohen’s kappa coefficient (κ), interpreted per Landis and Koch criteria. A *p*-value < 0.05 was considered statistically significant.

## RESULTS

### Baseline characteristics

A total of 357 patients with a prior cytological diagnosis of AUS who subsequently underwent thyroidectomy were included (**Table 1**). The cohort was predominantly female (n = 293, 82.1%), with a mean age of 46.6 ± 13.8 years (range: 17–87). Final histopathological analysis confirmed malignancy in 156 cases (43.7%) and benign findings in 201 (56.3%). In univariate analysis (**Table 2**), nuclear atypia was significantly more frequent in malignant cases (84.6%) compared with benign cases (49.3%) (*p* < 0.001). Malignant nodules more commonly exhibited high-risk ultrasonographic features, including solid composition (89.1% vs. 58.2%), hypoechogenicity (73.1% vs. 23.4%), irregular margins (45.5% vs. 6.0%), suspicious echogenic foci (54.5% vs. 15.9%), and a taller-than-wide shape (17.9% vs. 0.5%) (all *p* < 0.001). Nodules smaller than 10 mm were significantly more frequent in malignant cases (42.3%) than benign ones (12.9%) (*p* < 0.001). Additionally, a history of repeat biopsy was more common among malignant cases (75.6% vs. 65.2%; *p* = 0.033). All three risk stratification systems (ACR-TIRADS, EU-TIRADS, and K-TIRADS) were significantly associated with malignancy (*p* < 0.001).

**Table 1.** Baseline characteristics of the study population

Variable	Cohort (n = 357)
Sex, n (%)	
Female	293 (82.1)
Male	64 (17.9)
Age, mean ± SD (min–max)	46.6 ± 13.8 (17–87)
Surgical diagnosis, n (%)	
Benign	201 (56.3)
Malignant	156 (43.7)
History of re-FNAB, n (%)	249 (69.7)
Cytology	
AUS-N	231 (64.7)
AUS-O	126 (35.3)
Ultrasound features of the nodule	
Size (mm), n (%)	
<10	92 (25.8)
10–20	140 (39.2)
20–40	90 (25.2)
>40	35 (9.8)
Composition, n (%)	
Solid	256 (71.7)
Mixed (solid-cystic)	97 (27.2)
Spongiform	3 (0.8)
Completely cystic	1 (0.3)
Echogenicity, n (%)	
Anechoic	1 (0.3)
Iso-hyperechoic	195 (54.6)
Slightly hypoechoic	136 (38.1)
Markedly hypoechoic	25 (7.0)
Margin, n (%)	
Regular	274 (76.8)
Irregular	83 (23.2)
Extrathyroidal extension, n (%)	3(0.8)
Echogenic focus, n (%)	
None/comet-tail artifact	240 (67.2)
Macrocalcifications	29 (8.1)
Peripheral calcifications	7 (2.0)
Microcalcifications	81 (22.7)
Shape, n (%)	
Wider-than-tall	328 (91.9)
Taller-than-wide	29 (8.1)
Classifications	
ACR-TIRADS, n (%)	
ACR-TIRADS 1	4 (1.1)
ACR-TIRADS 2	64 (17.9)
ACR-TIRADS 3	89 (24.9)
ACR-TIRADS 4	111 (31.1)
ACR-TIRADS 5	89 (24.9)
EU-TIRADS, n (%)	
EU-TIRADS 2	4 (1.1)
EU-TIRADS 3	165 (46.2)
EU-TIRADS 4	53 (14.8)
EU-TIRADS 5	135 (37.8)
K-TIRADS, n (%)	
K-TIRADS 2	5 (1.4)
K-TIRADS 3	167 (46.8)
K-TIRADS 4	86 (24.1)
K-TIRADS 5	99 (27.7)

SD: Standard deviation; re-FNAB: repeat fine-needle aspiration biopsy; AUS-N: atypia of undetermined significance - nuclear atypia; AUS-O: atypia of undetermined significance - other atypia; ACR-TIRADS: American College of Radiology Thyroid Imaging Reporting and Data System; EU-TIRADS: European Thyroid Association Thyroid Imaging Reporting and Data System; K-TIRADS: Korean Thyroid Imaging Reporting and Data System.

**Table 2.** Univariate analysis of clinical and ultrasound predictors of malignancy in patients with AUS cytology

Variable	Benign (n = 201)	Malignant (n = 156)	p-value
Female sex, n (%)	168 (83.6)	125 (80.1)	0.399
Age (years), mean ± SD	47.6 ± 13.1	45.3 ± 14.6	0.119
History of re-FNAB, n (%)	131 (65.2)	118 (75.6)	0.033
AUS-N, n (%)	99 (49.3)	132 (84.6)	<0.001
Ultrasound features			
Nodule size <10 mm, n (%)	26 (12.9)	66 (42.3)	<0.001
Solid composition, n (%)	117 (58.2)	134 (89.1)	<0.001
Hypoechogenicity, n (%)	47 (23.4)	114 (73.1)	<0.001
Irregular margin, n (%)	12 (6.0)	71 (45.5)	<0.001
Suspicious echogenic foci, n (%)	32 (15.9)	85 (54.5)	<0.001
Taller-than-wide shape, n (%)	1 (0.5)	28 (17.9)	<0.001
ACR-TIRADS, n (%)			
ACR-TIRADS 1	4 (2.0)	0	<0.001
ACR-TIRADS 2	59 (29.4)	5 (3.2)	
ACR-TIRADS 3	77 (38.3)	12 (7.7)	
ACR-TIRADS 4	56 (27.9)	55 (35.3)	
ACR-TIRADS 5	5 (2.5)	84 (53.8)	
EU-TIRADS, n (%)			
EU-TIRADS 2	4 (2.0)	0	<0.001
EU-TIRADS 3	144 (71.6)	21 (13.5)	
EU-TIRADS 4	36 (17.9)	17 (10.9)	
EU-TIRADS 5	17 (8.5)	118 (75.6)	
K-TIRADS, n (%)			
K-TIRADS 2	5 (2.5)	0	<0.001
K-TIRADS 3	144 (71.6)	23 (14.7)	
K-TIRADS 4	40 (19.9)	46 (29.5)	
K-TIRADS 5	12 (6.0)	87 (55.8)	

re-FNAB: Repeat fine-needle aspiration biopsy; AUS-N: atypia of undetermined significance - nuclear atypia; ACR-TIRADS: American College of Radiology Thyroid Imaging Reporting and Data System; EU-TIRADS: European Thyroid Association Thyroid Imaging Reporting and Data System; K-TIRADS: Korean Thyroid Imaging Reporting and Data System.

### Regression analyses

A binomial logistic regression model was developed to identify independent predictors of malignancy (**Table 3**). The overall model was statistically significant (*p* < 0.001) and demonstrated strong discriminative capacity (Nagelkerke R^2^ = 0.607). No evidence of multicollinearity was observed; all variance inflation factors were below 1.25. After adjustment for other variables, the strongest independent predictor of malignancy was a taller-than-wide shape (OR = 17.95, 95% CI: 2.11–152.46;* p* = 0.008). Presence of suspicious echogenic foci (OR = 9.72, 95% CI: 4.34–21.75;* p* < 0.001) and irregular or extrathyroidal margins (OR = 8.43, 95% CI: 3.70–19.20;* p* < 0.001) were also strongly associated with malignancy. Nuclear atypia (OR = 4.52, 95% CI: 2.29–8.92;* p* < 0.001), solid composition (OR = 2.44, 95% CI: 1.12–5.31;* p* = 0.024), and hypoechogenicity (OR = 2.46, 95% CI: 1.27–4.75;* p* = 0.008) remained significant predictors. Nodule size was not independently associated with malignancy.

**Table 3.** Binomial logistic regression model predicting malignancy

Predictor	β	SE	Z	p-value	OR (95% CI)
Shape (taller-than-wide)	2.89	1.09	2.65	0.008	17.95 (2.11–152.46)
Echogenic focus (peripheral calcification or microcalcifications)	2.27	0.41	5.53	<0.001	9.72 (4.34–21.75)
Margin (irregular/extrathyroidal)	2.13	0.42	5.08	<0.001	8.43 (3.70–19.20)
Nuclear atypia (present)	1.51	0.35	4.35	<0.001	4.52 (2.29–8.92)
Echogenicity (hypoechoic)	0.90	0.34	2.67	0.008	2.46 (1.27–4.75)
Composition (solid)	0.89	0.40	2.26	0.024	2.44 (1.12–5.31)
Intercept	−3.45	0.45	−7.73	<0.001	0.03 (0.01–0.08)

Model summary: Nagelkerke R^2^ = 0.615; Overall model: *p* < 0.001. β: Beta coefficient; SE: standard error; OR: odds ratio; CI: confidence interval.

### Development of the new classification system

A point-based AUS-MRS was developed using β coefficients from the final multivariable logistic regression model (**Table 4**). Each variable’s coefficient was divided by the smallest statistically significant β (composition: 0.89) to calculate a point ratio, which was rounded to assign whole-number weights. The final scoring system included six predictors: taller-than-wide shape (6 points), high-risk echogenic foci (5 points), irregular or extrathyroidal margins (5 points), nuclear atypia (3 points), hypoechogenicity (2 points), and solid composition (2 points). Total risk scores ranged from 0 to 23 points.

**Table 4.** Derivation of point-based risk scoring system AUS-MRS from model coefficients

Predictor	β	Point ratio	Assigned points
Shape (taller-than-wide vs. wider-than-tall)	2.89	(2.89/0.89) = 3.25	6
Echogenic focus (high vs. low risk)	2.27	(2.27/0.89) = 2.55	5
Margin (irregular/extrathyroidal extension vs. regular)	2.13	(2.13/0.89) = 2.39	5
Nuclear atypia (present vs. absent)	1.51	(1.51/0.89) = 1.70	3
Echogenicity (hypoechoic vs. non-hypoechoic)	0.90	(0.90/0.89) = 1.01	2
Composition (solid vs. not solid)	0.89	(reference)	2

β: Beta coefficient.

**Figure 1.**
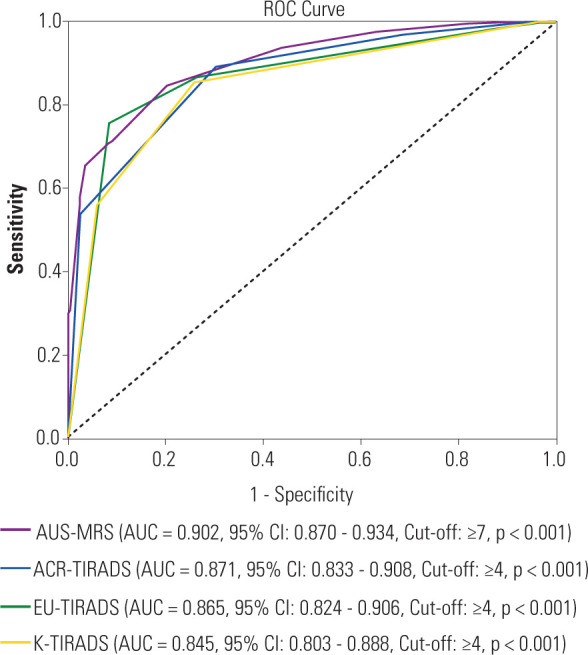
Receiver operating characteristic curve comparing the diagnostic performance of AUS-MRS and three established classification systems: ACR-TIRADS, EU-TIRADS, and K-TIRADS.

**Figure 1** presents the ROC curves comparing the diagnostic performance of AUS-MRS with three established ultrasound-based classification systems (ACR-TIRADS, EU-TIRADS, and K-TIRADS). The AUS-MRS yielded the highest discriminative power, with an AUC of 0.902 (95% CI: 0.870–0.934), followed by ACR-TIRADS (AUC = 0.871, 95% CI: 0.833–0.908), EU-TIRADS (AUC = 0.865, 95% CI: 0.824–0.906), and K-TIRADS (AUC = 0.845, 95% CI: 0.803–0.888). Pairwise comparison of AUCs via the DeLong test indicated that AUS-MRS had significantly higher diagnostic performance than ACR-TIRADS (*p* = 0.002), EU-TIRADS (*p* = 0.007), and K-TIRADS (*p* < 0.001). To minimize overfitting, internal validation using a split-sample approach was performed, with the AUS-MRS showing excellent discriminative performance in the validation cohort (AUC = 0.911, 95% CI: 0.854–0.968).

Optimal cut-off values for malignancy prediction, determined by the Youden index, were 7 for AUS-MRS and ≥4 for all TIRADS-based systems. All scoring systems demonstrated statistically significant discrimination between benign and malignant nodules (*p* < 0.001). AUS-MRS was further stratified into two risk categories: low-risk (<7 points) and high-risk (≥7 points). The malignancy rate increased markedly between these categories: 13.0% in the low-risk group and 76.3% in the high-risk group (**Table 5**).

**Table 5.** AUS Malignancy risk scoring system

Predictor	Score
Shape (taller-than-wide)	6
Echogenic focus (peripheral calcification or microcalcifications)	5
Margin (irregular or extrathyroidal extension)	5
Nuclear atypia (present)	3
Echogenicity (hypoechoic)	2
Composition (solid)	2
Total score range	0–23
Risk categories	Malignancy rate (%)
Low (<7 points)	13.0
High (≥7 points)	76.3

### Comparison of the classification systems

**Figure 2.**
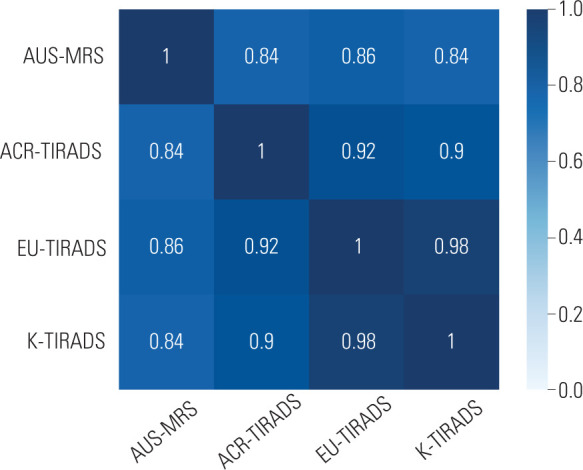
Heatmap illustrating the concordance of risk stratification among the classification systems. The values represent Cohen’s kappa coefficients for inter-rater agreement, where a value of 1.00 indicates perfect agreement. The color intensity corresponds to the strength of agreement, with darker blue indicating a higher kappa coefficient.

To compare diagnostic performance and agreement, all classification systems were dichotomized using clinically relevant cut-off values: ≥4 for ACR-, EU-, and K-TIRADS, and ≥7 for AUS-MRS, as established by ROC curve analysis and the Youden index. Pairwise Cohen’s kappa analyses demonstrated near-perfect agreement among the three conventional TIRADS systems, with the highest concordance between EU-TIRADS and K-TIRADS (κ = 0.98), followed by EU-TIRADS and ACR-TIRADS (κ = 0.92), and ACR-TIRADS and K-TIRADS (κ = 0.90). AUS-MRS showed slightly lower, though still high, levels of agreement with EU-TIRADS (κ = 0.86), K-TIRADS (κ = 0.84), and ACR-TIRADS (κ = 0.84) (**Figure 2**).

Diagnostic performance metrics for AUS-MRS and the three conventional systems are presented in **Table 6**. AUS-MRS yielded the highest diagnostic odds ratio (DOR = 21.8), with balanced sensitivity (84.6%) and specificity (79.6%). ACR-TIRADS achieved the highest sensitivity (89.1%) but lower specificity (69.7%). EU-TIRADS and K-TIRADS provided more balanced performance, with specificities of 73.6% and 74.1%, respectively. When AUS-N was incorporated into each TIRADS system, specificity increased notably in all models (to 85.1% for ACR-TIRADS, 86.6% for EU-TIRADS, and 86.1% for K-TIRADS), albeit with a concurrent reduction in sensitivity. The overall diagnostic odds ratios remained relatively unchanged.

**Table 6.** Diagnostic performance of the classification systems in predicting thyroid nodule malignancy

Classification system	Cutt-off	Total case	Sensitivity	Specificity	LR+	LR-	DOR
			(%)				
AUS-MRS	≥7	173 (48.5)	84.6	79.6	4.15	0.19	21.8
ACR-TIRADS	≥4	200 (56.0)	89.1	69.7	2.94	0.16	18.4
EU-TIRADS	≥4	188 (52.7)	86.5	73.6	3.28	0.18	18.2
K-TIRADS	≥4	185 (51.8)	85.3	74.1	3.29	0.20	16.7
ACR-TIRADS with AUS-N	≥4	149 (41.7)	76.3	85.1	5.12	0.28	18.3
EU-TIRADS with AUS-N	≥4	142 (39.8)	73.7	86.6	5.5	0.30	18.3
K-TIRADS with AUS-N	≥4	141 (39.5)	72.4	86.1	5.2	0.32	16.3

LR+: Positive likelihood ratios; LR−: negative likelihood ratios; DOR: diagnostic odds ratios; AUS-MRS: atypia of undetermined significance malignancy risk scoring; ACR-TIRADS: American College of Radiology Thyroid Imaging Reporting and Data System; EU-TIRADS: European Thyroid Association Thyroid Imaging Reporting and Data System; K-TIRADS: Korean Thyroid Imaging Reporting and Data System; AUS-N: atypia of undetermined significance - nuclear atypia.

## DISCUSSION

The TBSRTC has provided a standardized framework for the cytological evaluation of thyroid nodules; however, the management of nodules classified as AUS remains a significant clinical challenge. The ambiguity surrounding the AUS category is further compounded by the inconsistency in malignancy rates reported across studies, reflecting the heterogeneous nature of this cytological classification. In clinical settings where molecular testing is not readily available, a repeat FNAB is often recommended for patients with an initial AUS diagnosis (^21^,^22^). Nevertheless, approximately 30–40% of these nodules remain within the AUS category even after repeat FNAB, thereby complicating clinical decision-making (^23^,^24^).

Recent studies have proposed alternative strategies to improve malignancy risk prediction, such as subclassification of AUS based on nuclear atypia and evaluation of suspicious ultrasonographic features (^9^–^12^,^15^–^18^,^25^). Building on these approaches, we hypothesized that integrating cytological subgroups with key sonographic parameters could yield a more accurate method for predicting malignancy in AUS-diagnosed nodules. In this retrospective study, a novel malignancy risk stratification model (i.e., the AUS-MRS) was developed and validated by combining cytological subclassification (AUS-N vs. AUS-O) with sonographic findings. The findings indicated that AUS-MRS was associated with higher diagnostic performance than three other conventional ultrasound-based risk classification systems (ACR-TIRADS, EU-TIRADS, and K-TIRADS) for malignancy prediction. This highlights the importance of integrating cytological atypia and ultrasonographic risk features for a more refined and clinically actionable malignancy risk assessment in AUS-diagnosed nodules.

Ultrasonographic features of thyroid nodules are among the most extensively studied predictors of malignancy, particularly in nodules classified as AUS. In this study, the predictive value of key US parameters for malignancy was comprehensively evaluated. Consistent with previous reports, the presence of microcalcifications, hypoechogenicity, and irregular or extrathyroidal margins were identified as independent predictors of malignancy (^26^–^28^). Notably, the “taller-than-wide” morphology emerged as one of the most powerful statistical predictors (OR = 17.95), reaffirming the diagnostic significance of this specific sonographic feature. Furthermore, in line with the third edition (2023) of the TBSRTC, the AUS category was subclassified into nuclear atypia (AUS-N) and other atypia (AUS-O), allowing for a more nuanced clinical assessment. The current analysis demonstrated that the AUS-N subgroup was an independent and statistically significant predictor of malignancy (OR = 4.52), corroborating the findings of Valderrabano and cols. (^18^), who emphasized the diagnostic significance of nuclear atypia in indeterminate thyroid cytology, and Glass and cols. (^29^), who advocated for AUS specimen subclassification based on the type of atypia.

In the present study, the AUS-MRS, a novel malignancy risk scoring system was developed using independent predictors identified via multivariable logistic regression based on sonographic parameters and cytological subclassification. Integration of nuclear atypia with high-risk ultrasound features allowed for a scoring system that stratified patients into two categories: low risk (<7 points) and high risk (≥7 points). The AUS-MRS demonstrated superior diagnostic accuracy compared to current ultrasound-based classification systems. A distinctive strength of AUS-MRS is the quantitative integration of nuclear atypia as an independent predictor, to which 3 points were assigned. In contrast, current ultrasound-based systems (i.e., ACR-TIRADS, EU-TIRADS, and K-TIRADS) do not include nuclear atypia in their algorithms.

Yoo and cols. (^16^) reported that categorizing thyroid nodules with AUS cytology using updated US risk stratification systems, particularly when combined with cytological subtyping, may facilitate the determination of the most appropriate management strategy for patients. To address this gap, we conducted additional analyses by incorporating nuclear atypia as a binary variable (present/absent) into each conventional TIRADS system. Although this modification led to a notable increase in specificity (>85%), it resulted in decreased sensitivity, and overall diagnostic accuracy did not significantly improve. These findings suggest that binary incorporation of nuclear atypia offers limited added value, whereas weighted integration (as in the AUS-MRS) provides a more balanced and effective risk prediction when combined with sonographic features. Accordingly, cytological subclassification should not be considered merely as a supplementary factor, but rather a structural component in malignancy risk models designed for AUS-diagnosed nodules.

Clinically, the AUS-MRS should not be viewed as a stand-alone determinant for managing AUS-diagnosed thyroid nodules, but rather as a complementary risk stratification tool to support existing clinical approaches. In particular, in clinical settings where high-quality US evaluation and experienced cytopathological assessment, AUS-MRS may enhance clinical decision-making. By integrating cytological subclassification (especially nuclear atypia) with sonographic risk features, the AUS-MRS can help contextualize malignancy risk within the heterogeneous AUS category. In this framework, a cut-off value of ≥7 identifies a subgroup of AUS nodules with substantially increased malignancy risk, in whom closer follow-up, repeat biopsy, molecular testing, or earlier surgical intervention may be warranted based on clinical context and patient-specific factors. Conversely, lower AUS-MRS scores may favor more conservative management, including surveillance or deferred intervention, particularly in the absence of other high-risk clinical features.

This study has several limitations. First, the study population consisted exclusively of patients who underwent thyroidectomy following AUS cytology diagnosis, representing a preselected higher-risk cohort. This design introduces verification (work-up) bias, as analyses were limited to surgically treated nodules. As a result, the observed malignancy rate was higher than that reported in unselected or conservatively managed AUS populations, despite noninvasive follicular thyroid neoplasm with papillary-like nuclear features being classified as benign, and diagnostic performance metrics may have consequently been overestimated. Therefore, our findings should be interpreted in the context of surgically treated AUS nodules and may not be directly generalizable to all AUS cases. Second, the single-center and retrospective design of the study introduced potential selection and observation bias. Additionally, the AUS-MRS was not directly compared with molecular diagnostic methods, thus precluding a direct performance comparison.

Despite all US examinations being carried out by experienced endocrinologists, it is inherently operator-dependent and subjective. Consequently, the absence of interobserver agreement analysis for sonographic features constitutes a further significant limitation. Moreover, some predictors demonstrated wide confidence intervals, likely reflecting the low prevalence of certain high-risk sonographic features rather than model instability, and should be interpreted with caution. The diagnosis of AUS itself remains a subjective and interpretative category, with known interobserver variability among cytopathologists. Cytological specimens in this study were originally diagnosed by different pathologists and subsequently re-evaluated by an experienced cytopathologist who was blinded to the histopathological outcomes using the updated 2023 TBSRTC criteria. While this second assessment improved diagnostic consistency and confirmation of AUS categorization, it could not entirely eliminate pathologist-dependent variability. Consequently, the lack of full standardization—an inherent feature of AUS-based and ultrasound-driven studies—should be considered when applying and interpreting the AUS-MRS in routine clinical practice. More consistent application may be achieved in clinical settings with high-quality ultrasonography and experienced cytopathology, albeit reproducibility across operators and centers should not be overlooked. Although internal validation was performed, the absence of external validation is an additional limitation of this study.

The generalizability, reproducibility, and robustness of AUS-MRS across different populations and clinical settings remain to be established. Nevertheless, this study introduces the first scoring model integrating both cytological subclassification (particularly nuclear atypia) and essential sonographic features for malignancy risk stratification in AUS-diagnosed thyroid nodules. In this respect, the AUS-MRS is an innovative and comprehensive tool with the potential to enhance clinical decision-making. To validate and generalize our findings, prospective multicenter studies involving more diverse populations are warranted.

In conclusion, the AUS-MRS system was shown to improve malignancy prediction by integrating nuclear atypia with high-risk ultrasonographic features and was associated with higher diagnostic performance than existing TIRADS classification systems. Hence, this scoring model has the potential to serve as a valuable instrument to facilitate clinical decision-making in patients diagnosed with AUS. Nevertheless, external validation in larger, prospective, multicenter cohorts is necessary to confirm its generalizability and clinical utility.

## Data Availability

datasets related to this article will be available upon request to the corresponding author.
